# Detection of IL10-producing B cell (B10) in adenoids of atopic children with adenoidal hypertrophy

**DOI:** 10.1186/s13052-018-0471-3

**Published:** 2018-02-27

**Authors:** Chiara Valsecchi, Sara Carlotta Tagliacarne, Ilaria Brambilla, Catherine Klersy, Marco Benazzo, Lorenza Montagna, Dimitri Poddighe, Giorgio Ciprandi, Gian Luigi Marseglia, Amelia Licari, Annamaria Castellazzi

**Affiliations:** 1 Department of Clinical Surgical Diagnostic and Pediatric Sciences, University of Pavia and Fondazione IRCCS Policlinico San Matteo, P.le Golgi 19, 27100 Pavia, Italy; 2 Department of Clinical Surgical Diagnostic and Pediatric Sciences, University of Pavia, Pavia, Italy; 3 Department of Biometry and Statistics, Fondazione IRCCS Policlinico San Matteo, Pavia, Italy; 4 Department of Otolaryngology, University of Pavia and Fondazione IRCCS Policlinico S. Matteo, Pavia, Italy; 5 Department of Internal Medicine, Ospedale Policlinico S. Martino, Genoa, Italy

**Keywords:** Adenoids, B regulatory cells, Atopy

## Introduction and background

Regulatory cells are important for maintaining immunological homeostasis and tolerance to antigens, including self-antigens. Even though in the majority of autoimmune diseases and inflammatory processes, B cells generally play an important role due to their capacity to secrete antibodies, it has emerged that different B cell subsets may be able to down regulate immune responses, especially in experimental autoimmune disease models. Moreover, it has been demonstrated that B cells are able to drive T cell differentiation in favor of a regulatory phenotype (regulatory T cells, Treg) both in mice and humans [1].

In several studies the presence of Breg cells is often related to the development of chronic inflammation or autoimmune disease, but their pathogenic involvement is not completely understood. Researchers have described many B cell subsets with regulatory functions in mice, which are characterized by different surface markers, such as CD5^+^B-1a cells, CD19^hi^CD1d^hi^CD5^+^ B cells, or marginal zone (MZ) B cells [1]. Some studies have also suggested that human IL-10-producing B10 cells include CD24^hi^CD38^hi^ and CD24^hi^CD27^+^ B cells, which have been identified by their capacity to produce IL10 after appropriate stimulation [2].

However, Breg cells represent an heterogeneous group of immunosuppressive cells subset with distinct phenotypic and functional properties, that could play a role also in the induction of immune tolerance to allergens. Breg-derived IL10 is involved not only in inhibition of Th1 polarization, but also in preventing Th2 responses and its expression in mucosal environment is important for the generation of immunological tolerance. Moreover, allergic disorders are characterized by an imbalance of immune response, typically defined by type 2 response and by a functional defect of Treg (3). In particular, allergic inflammation is mediated and regulated by allergen-specific Th2 cells that induce B cells to produce IgG1 and IgE by secreting IL-4. Moreover, Th2 cells also recruit eosinophils via IL-5 production and directly act on epithelial cells and smooth muscle cells through IL-13 production*.* The development of the allergic environment, for example in asthma, could increase germinal center B cell numbers and MHC II and CD23 expression on follicular mature B cells in lung, bronchial lymph nodes (bLN) and spleen and affect the maturation of regulatory B cell subsets [3].

It is not clear if the alteration in Bregs differentiation in germinal centers is a consequence of the establishment of an allergic environment or vice versa contributes to allergic pathogenesis. Anyway, there is evidence that Breg cells are involved in allergic diseases [4].

A recent review by Palomares and colleagues highlights the importance of IL10- and TFGβ- producing Breg cells because of their immunosuppressive properties during allergic reaction. These cell subsets are increased both in mice and humans in response to high-dose allergen exposure [4]. Bregs are able to promote the differentiation of functional Treg cells suppressing the activation of effector T cells. However, the phenotypes of Breg cells and their mechanisms of action related to allergic responses still need to be clearly defined.

The aim of the current study was to evaluate the presence of IL-10 competent B regulatory cells in the adenoids of pediatric patients undergoing adenoidectomy for adenoidal hypertrophy and recurrent upper airway infections. The study was also designed to identify possible surface markers of B10 cells.

## Materials and methods

Globally, 111 paediatric patients (62 males, 49 females, median age 5.5 years, 25th–75th 4.5–6.8) were examined for the detection of adenoidal B10 cells, according to the number of adenoidal mononuclear cells (AMCs) recovered after processing the surgical samples. Then, children were subdivided into two groups: 37 atopic and 74 non-atopic children. Atopic children were defined as those with a genetic tendency to develop allergic diseases such as asthma, allergic rhinitis and atopic dermatitis (eczema) according to the American Academy of Allergy, Asthma and Immunology (AAAAI) definition [5]. In particularly, in our study atopy was defined as the presence of one (or more) relative who was affected by an allergic disease, according to validated criteria [5] as we considered also in another previous study by Tagliacarne and colleagues [6].

AMCs from both groups of patients were “in vitro” stimulated with CpG 2006 and CD40L following the protocol of Iwata et al. [7], but after 48 h of stimulation, no differences in AMC response to in vitro stimulation were observed. Only after 72 h of in vitro incubation with CpG and CD40 L, was stimulation detectable.

AMCs were then labelled with monoclonal antibodies specific for some surface markers (CD19, CD1d^hi^, CD5, CD24^hi^, CD38^bright^) and intracellular staining (IL10) and the percentage of each marker was analyzed in flow cytometry.

Statistical analyses were performed using Stata 14.2 (StataCorp, College Station TX, USA). A 2-sided *p*-value < 0.05 was considered statistically significant; given the exploratory nature of the study no multiple test adjustments were used. Data were described as the mean and standard deviation (SD) or median and quartiles if continuous.

## Results

**Fig. 1:**
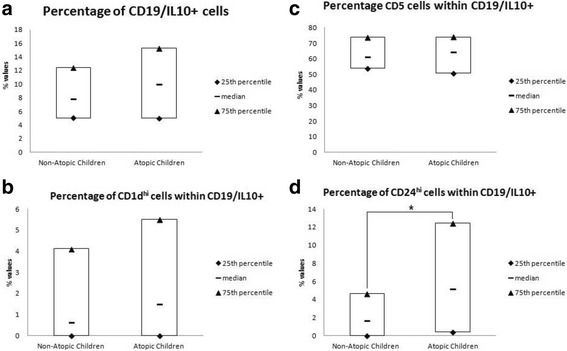
**a** Percentage of CD19/IL10+ cells expressed on the total of CD19+ cells; **b** percentage of CD1d^hi^ cells expressed on the total of CD19/IL10+ cells; **c** percentage of CD5+ cells expressed on the total of CD19/IL10+ cells; **d** percentage of CD24^hi^ cells analyzed within CD19/IL10+ cells expressed on the total of CD19/IL10+ cells. Comparisons between Atopic Children vs Non-Atopic Children: * *p* = 0.003 [Wilcoxon rank-sum (Mann-Whitney) test]

The CD19/IL10+ cells in adenoids were 8,50 [5–13.43] % of the total CD19+ cells. Notably, CD19/IL10+ cells frequency was higher in atopic than in non-atopic children, but this different is not statistically significant (*p* = 0.212) (Fig. 1a). In addition, we evaluated the expression of CD1d^hi^, CD5, CD24^hi^ on CD19/IL10+ cells. Atopic children had higher frequencies than non-atopic ones; in particular, there was significant difference (*p* = 0.003) for CD24^hi^ CD19/IL10+ cells (Fig. 1b–d).

## Discussion

So far, the presence of this cell subset was described only in murine models of autoimmune diseases [2]. In humans, they were detected in peripheral blood or in secondary lymphoid organs such as spleen, exclusively in autoimmune disorders [1].

Little is known about the involvement of B10 cells in the pathogenesis of chronic inflammatory processes or allergic diseases. Moreover, the presence of B10 was never described in literature in children in secondary lymphoid organs, such as adenoids, constitutively exposed to environmental antigens and allergens.

The persistence of an inflammatory microenvironment, due to recurrent upper airway infections or related to the atopic condition, could affect the susceptibility of AMCs to in vitro antigen stimulation, as we previously described [6].

To date, no clear phenotype, transcription factor, or lineage markers have been described for B10 cells.

A reduction in Breg percentages has been demonstrated only in peripheral blood of allergic patients and no evidence of their presence in secondary lymphoid organs such as tonsils and adenoids, particularly when exposed to environmental antigens has been reported [8].

Our study demonstrated that B10 cells are detectable in the adenoids of children with adenoidal hypertrophy and recurrent respiratory infections. Moreover, the percentage of CD19/IL10+ B cells was higher in atopic patients, probably due to the fact that their regulatory activity on other immunocompetent cells in lymphoid organs may be different than their regulatory activity in peripheral blood. Their suppressive role could be associated with IL10’s ability to inhibit chemokine and pro-inflammatory cytokine production and IL10’s ability to down regulate co-stimulatory molecule expression in APCs [8].

We also observed greater percentages of all investigated surface markers in allergic patients, significantly for CD24^hi^ CD19/IL10+ cells. CD24 controls T and B cell maturation and function and it could both improve and inhibit immune tolerance, regulating the efficiency of clonal deletion [9].

It is reasonable to think that the percentage of CD24high cells is related to the IL-10-producing B cells in theCD24high CD38^bright^ subpopulation percentage, but no detectable percentages in CD1d^hi^/CD5^+^ and CD24^hi^/CD38^bright^ double positive cells were observed in both groups (data not shown).

This could explain the association between this marker and higher incidence of autoimmunity, even thought it has not been demonstrated its involvement in allergy development. Moreover, in our study adenoids were removed as very hypertrophic as consequence of recurrent and severe local infections.

Recently, Komlósi and colleagues [10] detected also a mutual beneficial relationship between type 3 innate lymphoid cells (ILC3) and B cells in palatine tonsils of children. CD40+/ILC3 cells are involved in the in vivo differentiation of immature Bregs and are localized in tonsils. Noteworthy, tonsillar ILC3 frequency seems to be reduced in atopic patients. Due to their immune-modulatory capability in inducing Bregs differentiation, their scarce presence may induce a defective immune tolerance, characteristic of atopic subjects.

Therefore, our findings seem to disagree with that obtained by Komlósi and colleagues who reported lower Bregs frequency in atopic subjects. This conflicting finding could depend on different localization, structure, and function of adenoids and tonsils respectively. Palatine tonsils are localized near the entrances to the trachea and the esophagus, marked by a series of crypts, and exposed to both inhaled and alimentary antigens. Adenoids are situated posterior to the nasal cavity, do not possess crypts, have a lymphoepithelial structure, and are predominantly in contact with inhaled antigens and allergens. It is unclear if these characteristics could influence the functionality of immune B cells in tonsils and adenoids, but it has been already demonstrated that T follicular helper cells and B cells from adenoids have a more efficient functional response to bacterial antigens, than tonsillar cells [10].

Even though our current study is preliminary, it provides a first step in the characterization of the B10 cell subset in a secondary lymphoid organ, the adenoids, which are primarily involved in the immune response against microbes and environmental agents in the upper airways.

Although the data collected in this exploratory study point to important information in the characterization of adenoidal inflammation, we are aware of our study’s limitations. First of all, it was not possible to clearly identify a surface marker distinguishing this B cell subset, nor was it possible to define their functional properties. Due to the low number of cells recovered, purification of CD19/IL10^+^ cells was not possible; therefore we were unable to perform analyses of other surface markers, the detection of in vitro IL10 production or the evaluation of IL10 mRNA expression. In the future, we propose to characterize B10 cells in human adenoids, not only from the phenotypic point of view, using different surface markers, but also from a functional one, analysing the effect of this cell subset on TCD4+ functionality, proliferation and cytokine production in response to in vitro antigens, with a possible comparison of the results obtained in adenoids with those obtained in peripheral blood of the same patients.

However, the current study has some limitations: the lack of investigation concerning the kind of respiratory infections, the documentation of true allergy, and Bregs were evaluated only at adenoid tissue level.

Therefore, further studies should be addressed to respond to these unmet needs, in particular, to investigate possible relationships between the various cell populations located in different organs and in the blood.

## Conclusion

The current study provides the first evidence that B10 cells are present in adenoid tissue in children with adenoid hypertrophy probably as consequence of infectious inflammation. Atopy might amplify this phenomenon but, unfortunately, we couldn’t give an exhaustive explanation for the observed higher percentage, even though not statistically significant, of B10 regulatory cells in atopic patients, that seems to have an opposite trend compared to what was expected considering B10 role in the modulation of allergic responses. This could be probably due to the structural characteristics of adenoids, as suggested before, or to the different types of antigens and allergens to which the adenoids are exposed. We might speculate that the comprehension of B regulatory mechanisms of action could be useful in order to define B10 cells as a possible cellular marker for monitoring of allergic inflammation and a new therapeutic target for allergic diseases.

## References

[1] Kalampokis I, Yoshizaki A, Tedder TF (2013). IL-10-producing regulatory B cells (B10 cells) in autoimmune disease. Arthritis Res Ther.

[2] Jin G, Hamaguchi Y, Matsushita T, Hasegawa M, Le Huu D, Ishiura N, Naka K (2013). B cell linker protein expression contributes to controlling allergic and autoimmune diseases by mediating IL-10 production in regulatory B cells. J Allergy Clin Immunol.

[3] Annunziato F, Romagnani C, Romagnani S (2015). The 3 major types of innate and adaptive cell-mediated effector immunity. J Allergy Clin Immunol.

[4] Palomares O, Akdis M, Martín-Fontecha M, Akdis CA (2017). Mechanisms of immune regulation in allergic diseases: the role of regulatory T and B cells. Immunol Rev.

[5] Schussler E, Sobel J, Hsu J, Yu P, Meaney-Delman D, Grammer LC 3rd, Nowak-Wegrzyn A. Workgroup report by the joint task force involving American Academy of Allergy, Asthma & Immunology (AAAAI); food allergy, anaphylaxis, dermatology and drug allergy (FADDA) (adverse reactions to foods committee and adverse reactions to drugs, biologicals, and latex committee); and the Centers for Disease Control and Prevention botulism clinical treatment guidelines workgroup-allergic reactions to botulinum antitoxin: a systematic review. Clin Infect Dis 2017; 66(suppl_1):S65-S72. 10.1093/cid/cix827 PMC5850017 29293931

[6] Tagliacarne SC, Valsecchi C, Castellazzi AM, Licari A, Klersy C, Montagna L, Castagnoli R, Benazzo M, Ciprandi G, Marseglia GL (2015). Impact of passive smoke and/or atopy on adenoid immunoglobulin production in children. Immunol Lett.

[7] Iwata Y, Matsushita T, Horikawa M, Dilillo DJ, Yanaba K, Venturi GM, Szabolcs PM, Bernstein SH, Magro CM, Williams AD, Hall RP, St Clair EW, Tedder TF (2011). Characterization of a rare IL-10-competent B-cell subset in humans that parallels mouse regulatory B10 cells. Blood.

[8] Kim AS, Doherty TA, Karta MR, Das S, Baum R, Rosenthal P, Beppu A, Miller M, Kurten R, Broide DH (2016). Regulatory B cells and T follicular helper cells are reduced in allergic rhinitis. J Allergy Clin Immunol.

[9] Israel E, Kapelushnik J, Yermiahu T, Levi I, Yaniv I, Shpilberg O, Shubinsky G (2005). Expression of CD24 on CD19- CD79a+ early B-cell progenitors in human bone marrow. Cell Immunol.

[10] Komlósi ZI, Kovács N, van de Veen W, Kirsch A, Fahrner HB, Wawrzyniak M, Rebane A, Stanic B, Palomares O, Rückert B, Menz G, Akdis M, Losonczy G, Akdis CA (2017). Human CD40L-expressing type 3 innate lymphoid cells induce IL-10-producing immature transitional regulatory B cells. J Allergy Clin Immunol.

